# The impact of the ketogenic diet on the health of patients with metabolic syndrome: a scoping review

**DOI:** 10.3389/fnut.2026.1858811

**Published:** 2026-06-29

**Authors:** Martyna Winiarska, Dominika Wiśniewska, Michał Klimas, Dominik Jucha, Mateusz Szczupak, Jacek Kobak, Sabina Krupa-Nurcek

**Affiliations:** 1 Collegium Medicum, University of Rzeszów, Rzeszów, Poland; 2 Department of Anesthesiology and Intensive Care, Copernicus Hospital, Gdańsk, Poland; 3 Department of Otolaryngology, Faculty of Medicine, Medical University of Gdańsk, Gdańsk, Poland; 4 Department of Surgery, Faculty of Medicine, Collegium Medicum, University of Rzeszów, Rzeszów, Poland

**Keywords:** health, insulin resistance, ketogenic diet, ketosis, metabolic syndrome, nutrition therapy

## Abstract

**Introduction:**

The ketogenic diet, characterized by a very low carbohydrate intake and high fat consumption, has gained increasing attention as a potential strategy for managing metabolic syndrome.

**Methods:**

This scoping review aimed to summarize current evidence regarding the effects of the ketogenic diet on metabolic and clinical outcomes in patients with metabolic syndrome. A comprehensive literature search was conducted across PubMed, Scopus, Web of Science, EBSCO, Google Scholar, and the Cochrane Library, following PRISMA-ScR guidelines. Studies published between 2015 and 2025 in English were included.

**Results:**

The findings suggest that the ketogenic diet may contribute to weight reduction, improved insulin sensitivity, and beneficial changes in lipid profile, as well as reduced inflammation and improved glycemic control. However, concerns remain regarding long-term safety, potential micronutrient deficiencies, and variability in individual responses.

**Discussion:**

Overall, the ketogenic diet may represent a supportive therapeutic approach in metabolic syndrome, although further high-quality long-term studies are needed.

## 1 Introduction

The ketogenic diet is a specific nutrition model in which fats are the dominant source of energy and the supply of carbohydrates is drastically limited (1). As a result, the body goes into a state of ketosis, i.e., a metabolic adaptation involving the use of ketone bodies as the main energy fuel. From a clinical point of view, this diet has been used primarily in the treatment of drug-resistant epilepsy, especially in children, where its effectiveness in reducing the frequency of seizures has been documented. The mechanism of action is based on the stabilization of neuronal metabolism, the change in the balance of neurotransmitters and the improvement of mitochondrial function (2–5). In recent years, the ketogenic diet has become the subject of research in other areas of medicine, for example in the treatment of obesity and type 2 diabetes. Limiting carbohydrate intake improves glycemic control, increases insulin sensitivity and can lead to weight reduction, which is important in the treatment of metabolic syndrome (6). Its potential neuroprotective effects in neurodegenerative diseases such as Alzheimer’s and Parkinson’s disease are also being analyzed, although clinical evidence remains limited. In oncology, trials are being conducted to assess the impact of the ketogenic diet on cancer metabolism, but there are no clear recommendations (7). From a clinical perspective, the ketogenic diet also carries risks and limitations. The most common side effects include hyperlipidemia, kidney stones, constipation and vitamin and mineral deficiencies. It requires close monitoring by a doctor and dietician, especially in patients with diseases of the liver, pancreas or metabolic disorders related to the oxidation of fatty acids (8–10). The restrictiveness of the diet makes its long-term use difficult, and safety must be assessed individually. To sum up, the ketogenic diet is a therapeutic tool with documented efficacy in drug-resistant epilepsy and promising potential in other disease entities, but its use should only take place under controlled conditions, considering possible complications and the need to balance nutrients (11–13).

Metabolic syndrome, also called polymetabolic syndrome or syndrome X, is a clinical condition characterized by the simultaneous occurrence of several risk factors that mutually reinforce each other (14). It most often includes abdominal obesity, hypertension, carbohydrate metabolism disorders (e.g., increased fasting glucose levels or insulin resistance) and dyslipidemia, i.e., abnormal blood lipid levels – elevated triglycerides and decreased HDL cholesterol levels. To diagnose metabolic syndrome, criteria developed by various organizations are used, for example the National Cholesterol Education Program (NCEP ATP III) or the International Diabetes Federation (IDF) (15, 16). It is most often assumed that the presence of at least three out of five criteria allows for a diagnosis. These criteria include:

- Central obesity (waist circumference >94 cm in men and >80 cm in women in the European population).
- Increased blood pressure (≥130/85 mmHg or use of hypotensive drugs).
- Elevated fasting glucose levels (≥100 mg/dL or diabetes treatment).
- Increased triglyceride levels (≥150 mg/dL).
- Decreased HDL (<40 mg/dL in men, <50 mg/dL in women) (17, 18).

**Figure 1:**
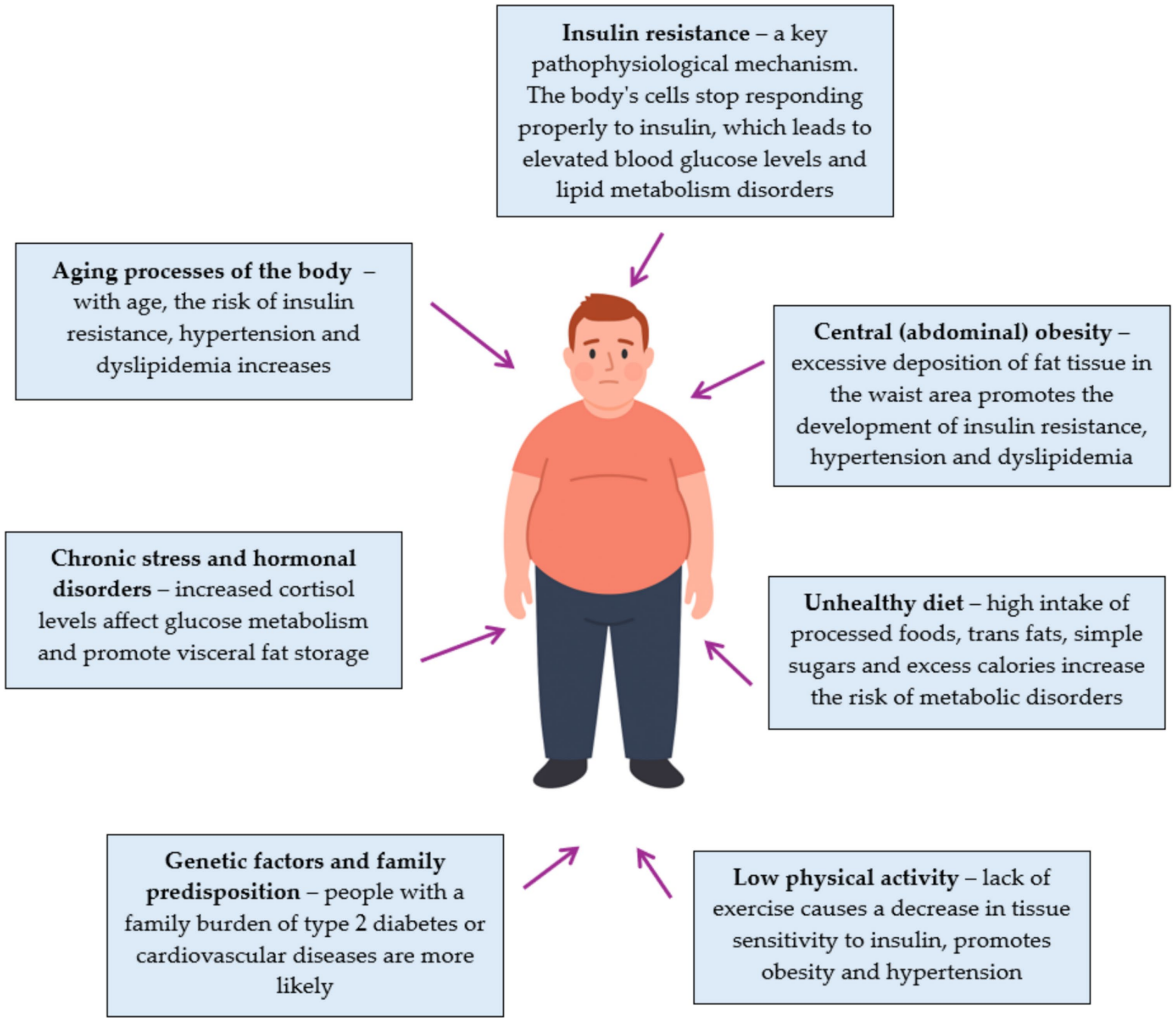
Top causes of metabolic syndrome. Source: authors’ own work.

Metabolic syndrome does not always give clear clinical symptoms, which is why it often develops insidiously. The most common is abdominal obesity, which is visible as excessive deposition of fat in the waist area. Patients may experience symptoms related to hypertension (headaches, dizziness, palpitations), carbohydrate metabolism disorders (excessive thirst, frequent urination, fatigue) or dyslipidemia, which is usually asymptomatic, but promotes the development of atherosclerosis (19). The greatest clinical importance of metabolic syndrome results from its complications. The risk of developing type 2 diabetes in people with metabolic syndrome is several times higher than in the general population. In addition, lipid disorders and hypertension promote the development of atherosclerosis, which leads to ischemic heart disease, myocardial infarction or stroke (19, 20). Metabolic syndrome is also associated with a higher risk of non-alcoholic fatty liver disease (NAFLD), polycystic ovary syndrome in women, and even certain cancers such as colorectal cancer or pancreatic cancer. Diagnosis of metabolic syndrome requires the evaluation of several clinical and biochemical parameters, and symptoms are often non-specific (21). The greatest threat is posed by complications – primarily cardiovascular diseases and type 2 diabetes. Therefore, early detection of metabolic syndrome and lifestyle modifications, including weight reduction, a healthy diet and regular physical activity, are crucial, which can significantly reduce the risk of serious health consequences (19, 22) (Figure 1).

### 1.1 Objectives and rationale

The aim of this review was to examine the available literature on the impact of a ketogenic diet on the health of patients with metabolic syndrome. Of particular interest were the factors that play a key role in the development of metabolic syndrome and the impact of a ketogenic diet on the health of these patients. The review question (RQ) for this review was: How does a ketogenic diet impact the health of patients with metabolic syndrome?

## 2 Materials and methods

### 2.1 Study design

We chose a scoping review approach because we wanted to create a map of concepts relevant to the phenomenon of metabolic syndrome and the ketogenic diet. Scoping reviews are a new methodological approach. Currently, there is little guidance on choosing between a systematic review and a scoping review when synthesizing evidence, particularly when the literature has not yet been comprehensively analyzed or is large, complex, or heterogeneous and not suitable for a more detailed systematic review (23). We conducted our scoping review according to the methods described in the Joanna Briggs Institute Methodology Manual for Scoping Reviews and using the recommendations in the Preferred Reporting Items for Systematic Reviews and Meta-analysis for Scoping Reviews (PRISMA-ScR) guidelines (24, 25).

### 2.2 Inclusion and exclusion criteria

To identify important aspects related to the phenomenon of the use of ketogenic diet in patients suffering from metabolic syndrome, we developed a research question that clearly defined the population, concept, and context of the review scope.

The inclusion criteria were as follows:

- Articles published in 2015–2025.
- Original articles (observational and randomized trials), meta-analyses, systematic and narrative reviews.
- Articles with access to the full text.
- English-language articles.
- Studies involving humans and animals.

The exclusion criteria included the following:

- Publications older than 10 years.
- Case reports, comments, letters to the editor, book chapters.
- No full-text article.
- Articles in a language other than English.

#### 2.2.1 Population

The review included studies reporting the use of a ketogenic diet in patients with metabolic syndrome. In this review, a ketogenic diet was defined as a diet characterized by a very low intake of carbohydrates and a high intake of fats, leading to a metabolic state called ketosis, in which the body draws energy from ketone bodies instead of glucose. Metabolic syndrome is defined as a condition in which there is insulin resistance or impaired glucose tolerance (impaired glucose tolerance or type 2 diabetes) and at least two of the following factors: increased blood pressure (≥140/90 mm Hg), increased triglyceride concentration (1.7 mmoL/L) and/or decreased HDL cholesterol levels <0.9 mmol\l in men and <1.0 mmol\l in women, central obesity (WHR > 0.9 in men and >0.85 in women or BMI > 30 kg\m^2^) and microalbuminuria (6, 11, 18, 19).

#### 2.2.2 Concept

The focus was on the impact of a ketogenic diet on patients with metabolic syndrome. The aim of the study was to assess the impact of a ketogenic diet on the health of patients with metabolic syndrome.

#### 2.2.3 Context

The studies to be included in the review included patients with metabolic syndrome.

#### 2.2.4 Types of studies

This review included a retrospective observational study of any design or methodology.

### 2.3 Search strategy

Three authors searched the following databases: PubMed (*n* = 58), Scopus (*n* = 72), EBSCO (*n* = 41), Web of Science (*n* = 49), Google Scholar (*n* = 40), and the Cochrane Library (*n* = 6). The Mozilla Firefox search engine was used for searching. The following keywords were used: “diet,” “ketogenic diet,” “metabolic syndrome,” “diet in metabolic syndrome”, “ketogenic diet in metabolic syndrome”. Many studies addressed dietary design in metabolic syndrome, which was not the purpose of this review. We entered keywords and their combinations using AND or OR operators. All publications were screened by title and abstract to exclude irrelevant items. Any discrepancies were resolved through consultation with five investigators, and at the end of the screening process, full consensus was reached on the articles to be included. The initial search ran from inception to August 7, 2025, and the final search ran until October 11, 2025.

### 2.4 Extraction of data

A data extraction form based on the JBI scoping review guidelines (24) was used and key information from the studies was included. Data extraction, which is referred to as “data plotting” in the scoping review (24), was performed by two independent reviewers. To identify relevant studies, we used the Population-Concept-Context (PCC) framework. Information extracted from the studies included: first author, year, country, study design, study objective, inclusion and exclusion criteria (PCC), outcomes, and findings. The authors performed the extraction using Microsoft Excel.

### 2.5 Critical appraisal process

A scoping review may include a review of current evidence without including a methodological assessment of the included studies (24).

### 2.6 Process for including publications to the review

**Figure 2:**
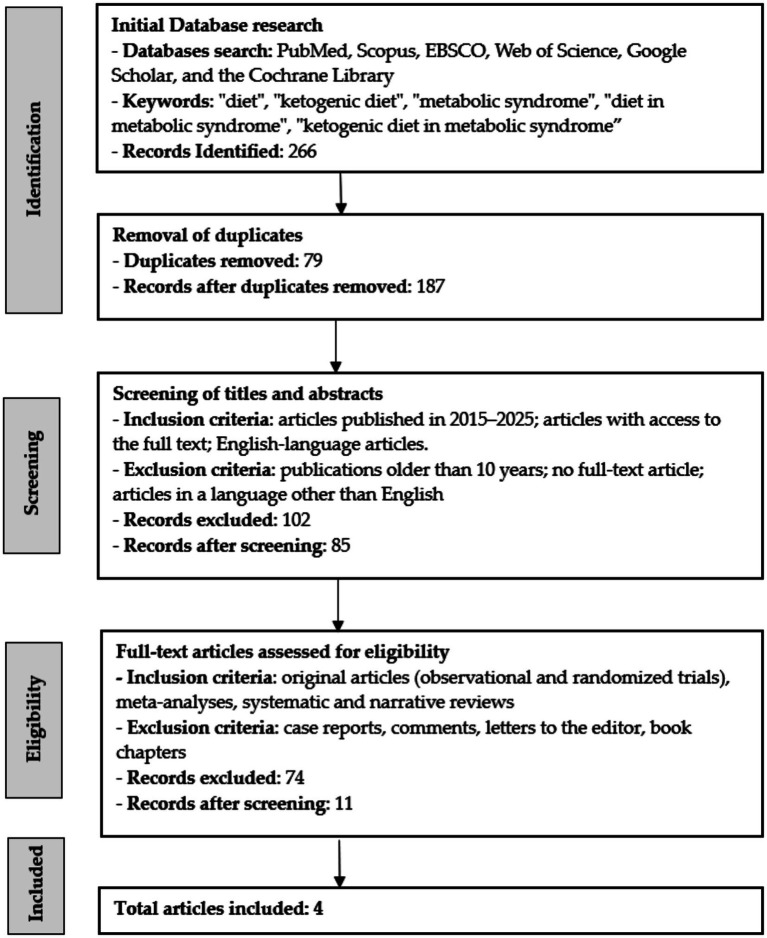
Literature search and selection flowchart for this review.

Our scoping review initially identified 266 articles, of which four were ultimately included in the review (Figure 2). After removing duplicates (*n* = 79), 187 articles remained. After reviewing the articles according to the inclusion and exclusion criteria (*n* = 102), 85 articles remained. Seventy-four publications lacked full text and were excluded, leaving 11 articles. Ultimately, after meeting all requirements, four publications were included in the review. Studies were conducted in Belgium (*n* = 1), the USA (*n* = 1), Bulgaria (*n* = 1), and China (*n* = 1). The results are presented in Table 1.

**Table 1:** Characteristics and findings of studies included in this review.

Author, year	Country	Participants	Findings
Delrue C. et al., 2025 (73)	Belgium	Patients with metabolic syndrome	Both intermittent fasting and the ketogenic diet independently show clinically significant benefits in improving metabolic parameters. Thanks to the ketogenic diet, there is a reduction in HbA1c, fasting glucose, body weight and triglycerides. The use of ketogenic diets and intermittent fasting increases insulin sensitivity, promote autophagy, reduce inflammation, and activate AMP-activated protein kinase (AMPK) while inhibiting the mammalian target of rapamycin (mTOR). The combination of intermittent fasting and the ketogenic diet may offer synergistic metabolic effects. Data on long-term safety and patient suitability remain limited.
Cooper M. A. et al., 2018 (74)	USA	Patients with metabolic syndrome	The implementation of the ketogenic diet has had a positive effect on insulin levels and affects the growth of nerve cells. A ketogenic diet reduces metabolic syndrome-induced allodynia and promotes peripheral nerve growth. Ketogenic diet may reduce symptoms of neuropathy.
Paskaleva I. N. et al., 2025 (75)	Bulgaria	Children with metabolic syndrome	A well-formulated short-term ketogenic diet is effective in treating obesity, metabolic syndrome, and related comorbidities, and can be part of a comprehensive approach for these patients. The ketogenic diet improves insulin sensitivity and regression of metabolic syndrome in a significant proportion of patients. Low-carbohydrate diet can be used as one of the possible dietary options in a comprehensive approach in children with obesity and metabolic syndrome.
Chen J. et al., 2025 (29)	China	Patients with metabolic syndrome	Future research should focus on the effects of KD on various aspects of MetS, especially its adaptation in different populations. Despite the increase in aldosterone, cardiovascular metabolic risks, including blood pressure and blood glucose levels, remained intact in the KD group. KD may play a potentially protective role in the treatment of MetS, especially for blood pressure and cardiovascular health. KD not only helps alleviate inflammation associated with MetS but also significantly improves metabolic indices, such as body weight, blood glucose, blood lipids, and blood pressure, thus providing an important adjunctive role in the treatment of MetS.

## 3 Components of the ketogenic diet that play a key role in metabolic syndrome

### 3.1 Fats

The ketogenic diet, which is based on a high supply of fats while limiting carbohydrates, plays a special role in modulating the body’s metabolic processes (2, 6). In the case of metabolic syndrome – a set of disorders including insulin resistance, visceral obesity, dyslipidemia and hypertension – fats become a key nutrient that can affect both health parameters and the risk of complications. The primary mechanism of action of fats in the ketogenic diet is their participation in the production of ketone bodies. In conditions of limited carbohydrate supply, the body enters a state of ketosis, in which fatty acids and their metabolites become the main source of energy (9–11). Ketone bodies, such as beta-hydroxybutyrate, have anti-inflammatory effects, improve insulin sensitivity and reduce oxidative stress, which is of direct importance in the treatment of metabolic syndrome. However, the type of fats consumed is of great importance. Unsaturated fats, especially monounsaturated and polyunsaturated fats, present in olive oil, avocados, nuts or oily fish, support a beneficial lipid profile (26). They lower triglyceride levels, increase HDL concentrations and can reduce the risk of atherosclerosis. In the context of metabolic syndrome, this is particularly important, as dyslipidemia is one of its main diagnostic elements. On the other hand, saturated fats, present in red meat or dairy products, in excess can lead to an increase in LDL cholesterol levels, which increases the risk of cardiovascular diseases (27). Therefore, the ketogenic diet should be composed in such a way that vegetable and fish fats dominate, and the proportion of saturated fats is limited. Omega-3 fatty acids, which have anti-inflammatory effects and support the functioning of the cardiovascular system, also play an important role. In metabolic syndrome, where chronic inflammation plays a key role in the development of insulin resistance and atherosclerosis, the presence of omega-3 in the ketogenic diet can significantly improve health parameters (28). Fats also affect the regulation of appetite and body weight. The consumption of fats in the ketogenic diet promotes the prolongation of satiety, which makes it easier to control the calorie content of meals and reduce body weight. Reducing the amount of body fat, especially visceral fat, is one of the most important factors improving the course of metabolic syndrome. Visceral tissue is metabolically active and secretes pro-inflammatory cytokines, which intensify insulin resistance (29, 30). The role of fats in the regulation of blood pressure cannot be overlooked either. A diet high in unsaturated fats can support blood vessel elasticity and improve endothelial function, which promotes lower blood pressure. Excess saturated fats, on the other hand, can have the opposite effect, increasing the risk of hypertension. Fats in the ketogenic diet play a multidimensional role in the context of metabolic syndrome. On the one hand, they are the main source of energy and induce a beneficial state of ketosis, which improves glucose-insulin metabolism (26, 31). On the other hand, the right selection of fats – with a predominance of unsaturated fats and the presence of omega-3 acids – supports weight reduction, improvement of lipid profile, lowering blood pressure and reducing inflammation. A properly balanced ketogenic diet can therefore be an effective tool to support the therapy of metabolic syndrome, provided that fat sources are consciously composed, and health parameters are monitored (32).

### 3.2 Protein

The ketogenic diet, whose main assumption is to limit the supply of carbohydrates and increase the proportion of fats in the daily menu, also assumes a moderate amount of protein. Although fats are the primary source of energy in this diet, proteins play a key role in maintaining metabolic balance, especially for people with metabolic syndrome (33). This syndrome includes the co-occurrence of insulin resistance, visceral obesity, dyslipidemia and hypertension, and an adequate supply of protein may support the improvement of many of these parameters (34). Protein in the ketogenic diet primarily has a building function, supporting the maintenance and regeneration of muscle mass. This is especially important in the process of reducing body fat, which often accompanies the keto diet. Loss of muscle mass could lead to a deterioration in metabolism and a decrease in the body’s ability to burn energy, so a moderate amount of protein (usually around 1–1.5 g/kg of body weight) allows you to maintain a balance between reducing body fat and maintaining muscle (34–36). In the context of metabolic syndrome, this is extremely important, as muscle mass supports insulin sensitivity and improves glucose metabolism. Another aspect is the effect of protein on appetite regulation and weight control. Protein intake increases satiety by stimulating the secretion of hormones such as peptide YY or GLP-1, which are responsible for controlling hunger. In practice, this means that people on a ketogenic diet feel less likely to feel the need to snack, which promotes a reduction in the calorie content of meals and weight loss (37). Reducing the amount of adipose tissue, especially visceral fat, is one of the most important factors improving the course of metabolic syndrome, because visceral fat secretes pro-inflammatory cytokines that intensify insulin resistance. Protein also has a significant effect on glucose-insulin metabolism. In the ketogenic diet, where the supply of carbohydrates is minimal, protein becomes an important element in stabilizing blood glucose levels. Amino acids can be used in the process of gluconeogenesis, i.e., the production of glucose in the liver, which allows it to maintain its appropriate concentration for tissues that require glucose, such as the brain or erythrocytes. At the same time, a moderate amount of protein prevents excessive fluctuations in blood sugar levels and supports the improvement of insulin sensitivity, which is crucial in the treatment of metabolic syndrome. The role of protein in the regulation of the lipid profile cannot be overlooked either (34, 38). Although fats are the main factor affecting cholesterol and triglyceride levels, an adequate supply of protein supports lipid metabolism and may contribute to lowering triglyceride levels. Combined with carbohydrate restriction, protein promotes improved lipid parameters, which reduces the risk of atherosclerosis and cardiovascular disease. It is worth noting that an excessive amount of protein in the ketogenic diet can be unfavorable (39). Too high a supply of amino acids leads to increased gluconeogenesis, which can make it difficult to maintain a state of ketosis. Therefore, the ketogenic diet is not a high-protein diet, but a moderate one in terms of the supply of this macronutrient. The optimal amount of protein allows you to maintain muscle mass, support appetite control, improve glucose and lipid balance, and promote weight reduction (33–36). Proteins in the ketogenic diet play a multidimensional role in the context of metabolic syndrome. They support the maintenance of muscle mass, regulate appetite, stabilize blood glucose levels, improve insulin sensitivity and support beneficial changes in the lipid profile. Thanks to these mechanisms, a moderate supply of protein in the ketogenic diet can be an important element in supporting the therapy of metabolic syndrome, provided that the diet is properly balanced and conducted under the supervision of a specialist (40, 41).

### 3.3 Micronutrients

The ketogenic diet, based on a high supply of fats, moderate amounts of protein and a very low carbohydrate content, focuses primarily on macronutrients. However, micronutrients – vitamins, minerals and trace elements – are equally important, as they play a key role in maintaining metabolic balance (42). In the case of metabolic syndrome, which includes insulin resistance, visceral obesity, dyslipidemia and hypertension, an adequate supply of micronutrients is essential to support metabolic processes and minimize the risk of complications. One of the most important elements in the ketogenic diet is magnesium, which is involved in more than 300 enzymatic reactions, including the regulation of glucose metabolism and the functioning of the cardiovascular system. Magnesium deficiency can intensify insulin resistance, increase blood pressure and promote heart rhythm disorders (42–44). In the ketogenic diet, due to the restriction of whole grains and fruits, the risk of magnesium deficiency is increased, so it is especially important to get it from nuts, seeds or green leafy vegetables. Another key micronutrient is potassium, which is responsible for regulating blood pressure, nerve conduction and muscle function. In metabolic syndrome, where hypertension is common, an adequate supply of potassium can support the reduction of blood pressure and the improvement of cardiovascular function. In the ketogenic diet, due to increased diuresis in the initial phase of ketosis, there is a loss of potassium, which can lead to muscle weakness and cramps. Therefore, it is recommended to consume avocado, spinach or salmon as sources of this element (45). Equally important is sodium, whose role in the ketogenic diet is different from that of traditional dietary recommendations. Carbohydrate restriction causes insulin levels to drop, and this, in turn, increases sodium excretion by the kidneys. Sodium deficiency can lead to symptoms such as fatigue, headaches or a drop in blood pressure. In the context of metabolic syndrome, a controlled supply of sodium is important – its excess promotes hypertension, but moderate amounts are necessary to maintain electrolyte balance in the ketogenic diet. Calcium also plays an important role, which supports bone health, but also participates in the regulation of blood pressure and lipid metabolism. Calcium deficiency can increase the risk of osteoporosis, as well as affect fat metabolism disorders. In a ketogenic diet, low-carbohydrate dairy products, such as ripened cheeses, can be a source of calcium (44, 46). The importance of B vitamins, which participate in energy metabolism and metabolism of carbohydrates, fats and proteins, cannot be overlooked. In metabolic syndrome, these vitamins support the improvement of nervous system function and the regulation of homocysteine levels, the excess of which increases the risk of cardiovascular disease. In the ketogenic diet, they are sourced from meat, eggs and green vegetables (47). A special role is played by vitamin D, the deficiency of which is common and associated with a higher risk of insulin resistance, obesity and cardiovascular diseases. Vitamin D supports the regulation of calcium-phosphate metabolism but also affects the functioning of the immune system and reduces inflammation, which is one of the mechanisms that intensify metabolic syndrome. In the ketogenic diet, its source is fatty fish, eggs and sun exposure. Antioxidants such as vitamin E, vitamin C and selenium are also important (48). In metabolic syndrome, oxidative stress plays a key role in the development of complications, so the presence of these micronutrients in the diet supports the neutralization of free radicals and reduces the risk of cellular damage. In the ketogenic diet, their sources are nuts, seeds, green vegetables and fish. In conclusion, micronutrients in the ketogenic diet play a fundamental role in the context of metabolic syndrome (47, 49). Magnesium, potassium and sodium support electrolyte balance and cardiovascular function, calcium and vitamin D take care of bone health and blood pressure regulation, B vitamins support energy metabolism, and antioxidants protect against oxidative stress. An adequate supply of micronutrients is essential for the ketogenic diet to be not only effective in reducing weight and improving glucose management, but also safe and supporting the long-term health of people with metabolic syndrome (50).

### 3.4 Carbohydrates

The ketogenic diet is a specific nutrition model in which the supply of carbohydrates is limited to a very low level – usually less than 50 g per day. Such a radical reduction in the amount of this macronutrient is crucial for metabolic mechanisms, especially in the case of people suffering from metabolic syndrome (51). This syndrome includes the co-occurrence of insulin resistance, visceral obesity, dyslipidemia and hypertension, and carbohydrate restriction in the ketogenic diet affects each of these elements. The primary role of carbohydrates in the body is to provide energy in the form of glucose. In a traditional diet, glucose is the main fuel for the brain and muscles, and its excess leads to increased insulin secretion. In the case of metabolic syndrome, where insulin resistance is one of the main disorders, an excessive supply of carbohydrates exacerbates the problem, causing further deposition of body fat and deterioration of glucose metabolism (52, 53). Restricting carbohydrates in the ketogenic diet reduces blood glucose, reduces insulin levels, and improves tissue sensitivity to this hormone. Thanks to this, it is possible to inhibit the progression of insulin resistance and reduce the risk of developing type 2 diabetes. A low supply of carbohydrates leads to the activation of an alternative energy pathway – the production of ketone bodies in the liver (54). In ketosis, the body gets its energy mainly from fats, and ketone bodies become fuel for the brain and muscles. This mechanism is of particular importance in the metabolic syndrome, as it reduces fluctuations in blood glucose levels and stabilizes energy management. As a result, people on a ketogenic diet experience less hunger, which promotes weight reduction (55). Carbohydrates in the ketogenic diet also play a role in regulating the lipid profile. Limiting their supply reduces the concentration of triglycerides in the blood, which is one of the diagnostic criteria for metabolic syndrome. At the same time, an increase in the HDL fraction, i.e., “good cholesterol,” which protects against atherosclerosis, is observed. A high supply of carbohydrates, especially simple and highly processed ones, has the opposite effect – it increases triglyceride levels and promotes visceral fat storage. An important aspect is also the impact of carbohydrate restriction on blood pressure (56). The ketogenic diet, by reducing insulin resistance and reducing body weight, promotes lower blood pressure. Excess carbohydrates, especially in the form of simple sugars, lead to sodium and water retention in the body, which increases the volume of circulating blood and raises blood pressure. Limiting their supply has the opposite effect – it supports electrolyte balance and improves the function of blood vessels. It is worth noting that carbohydrates in the ketogenic diet are not completely eliminated but limited to a minimum amount. Their source should be products with high nutritional value, such as non-starchy vegetables, which provide fiber, vitamins and minerals (53, 54, 57). Fiber plays a special role in regulating bowel function, supports blood glucose control and promotes cholesterol reduction. In the context of metabolic syndrome, the presence of fiber in the ketogenic diet is essential to minimize the risk of digestive disorders and support long-term health (58). Carbohydrates in the ketogenic diet play a limited role, but extremely important in the context of metabolic syndrome. Their reduction leads to improved insulin sensitivity, stabilization of glucose levels, reduction of triglycerides, increase in HDL fraction and reduction of body weight (59, 60). Thanks to this, the ketogenic diet can be an effective tool to support the therapy of metabolic syndrome, provided that carbohydrates are supplied from valuable sources, and the entire diet remains properly balanced and monitored by a specialist (61).

## 4 Controversies of the use of the ketogenic diet in metabolic syndrome

The use of a ketogenic diet in patients with metabolic syndrome may have short-term benefits, such as weight reduction or improved insulin sensitivity, but it is also associated with a number of potential dangers (62). The most important risks relate to lipid metabolism disorders, the risk of micronutrient deficiencies, burden on the liver and kidneys, as well as difficulties in maintaining a long-term diet. The ketogenic diet, which consists of a very low supply of carbohydrates and a high intake of fats, is increasingly used as a tool to support the therapy of metabolic syndrome. Carbohydrate restriction leads to lower glucose and insulin levels, which promotes improved insulin sensitivity and reduction of visceral fat (27, 37, 39). However, long-term use of this model of nutrition can be associated with serious health consequences. One of the main risks is hyperlipidemia, i.e., elevated levels of lipids in the blood. A high supply of fats, especially saturated fats, can lead to an increase in LDL cholesterol and triglycerides, which increases the risk of atherosclerosis and cardiovascular disease. Although an improvement in the lipid profile is observed in some patients, the body’s reaction is individual and not always predictable (30–33).

Another problem is the burden on the liver and kidneys. The liver is responsible for the production of ketone bodies, and long-term ketosis can lead to fatty liver and liver dysfunction. In turn, the kidneys are exposed to increased excretion of ketone bodies and electrolytes, which can promote dehydration, kidney stones and acid–base imbalances (63). Patients with metabolic syndrome often suffer from hypertension, so the risk of electrolyte disorders is particularly important. In the initial phase of the ketogenic diet, there is increased diuresis and loss of sodium, potassium and magnesium. This can lead to weakness, muscle cramps, heart rhythm disturbances or pressure drops. Micronutrient deficiencies are further exacerbated by the restriction of fruits, whole grains, and some vegetables, which are a natural source of vitamins and minerals (64). Glucose intolerance in the long term is also a significant threat. Studies indicate that chronic use of a ketogenic diet can disrupt insulin secretion and lead to a deterioration in carbohydrate metabolism. This means that after returning to a more varied diet, patients may experience difficulty maintaining stable glucose levels (65).

Dangers also include problems on the part of the digestive system. Dietary fiber restriction, resulting from a low supply of cereals and fruits, can lead to constipation, intestinal microbiota disorders and an increased risk of bowel disease. Metabolic syndrome often co-occurs with chronic inflammation, and microbiota disorders can further intensify this process (66, 67). In the initial phase of the diet, many patients experience the so-called “keto flu,” i.e., adaptation symptoms such as headaches, fatigue, nausea or irritability. Although they usually go away after a few days, they can be especially bothersome for people with metabolic syndrome, who often struggle with reduced exercise tolerance and chronic fatigue (65).

The psychological and practical aspects cannot be overlooked either. The ketogenic diet is restrictive and difficult to maintain in the long term. It requires the elimination of many products, which can lead to nutritional monotony, reduced quality of life and the risk of eating disorders. Patients with metabolic syndrome, who often need a permanent lifestyle change, may find it difficult to maintain such a rigorous nutrition model (62, 68). The use of the ketogenic diet in patients with metabolic syndrome carries the risk of hyperlipidemia, liver and kidney strain, electrolyte disorders, vitamin and mineral deficiencies, intestinal problems, and difficulties in maintaining the diet in the long term (69). Although it may improve metabolic parameters in the short term, its long-term safety raises serious doubts. Therefore, the ketogenic diet should only be used under the supervision of a specialist, with regular monitoring of lipid profile, liver and kidney function, and electrolyte balance (70–72) (Table 2).

**Table 2:** Summary of key factors of the ketogenic diet that influence the health of patients with metabolic syndrome.

Factor	Key findings	References
Fats	Excess visceral fat promotes insulin resistance, increased triglyceride levels and cholesterol imbalances, which increases the risk of type 2 diabetes and cardiovascular diseases. Healthy fats, such as omega-3 s, on the other hand, support the improvement of the blood lipid profile and may reduce the risk of metabolic syndrome.	(2, 6, 9–11, 26–32)
Protein	Their adequate supply supports weight control, improves tissue sensitivity to insulin and promotes the maintenance of normal blood glucose levels. Additionally, proteins may have a beneficial effect on the lipid profile, reducing the risk of cardiovascular complications associated with metabolic syndrome.	(16, 19, 33–41)
Micronutrients	Deficiencies of elements such as zinc, magnesium or iron disrupt hormonal and enzymatic balance, which promotes insulin resistance and an increased risk of type 2 diabetes. In turn, an adequate supply of micronutrients supports proper glucose and lipid metabolism, reducing the risk of cardiovascular complications.	(8, 11, 14, 42–50)
Carbohydrates	Excessive intake of simple sugars leads to insulin resistance, increased blood glucose levels and visceral fat deposition. Consequently, this increases the risk of type 2 diabetes and cardiovascular disease, which are the main components of metabolic syndrome.	(9, 12, 51–61)

## 5 Discussion

The results of this review indicate that the ketogenic diet may represent a promising strategy to support the treatment of metabolic syndrome, positively influencing key metabolic parameters such as body weight, insulin resistance, glycemia, lipid profile, and markers of inflammation. According to the analyzed studies, both short-term and medium-term ketogenic interventions led to improved clinical parameters in patients with metabolic syndrome, which is consistent with previous reports on the effectiveness of low-carbohydrate diets in metabolic disorders (6, 11). At the same time, however, the literature emphasizes the need for caution in assessing the long-term safety of this intervention, especially in the context of potential micronutrient deficiencies, changes in lipid metabolism and difficulties in maintaining a diet (8–10). In the study by Delrue et al., it has been shown that ketogenic diets and intermittent fasting can work synergistically, leading to improved insulin sensitivity, weight reduction, and decreased triglycerides. The mechanisms underlying these effects include activation of AMPK, inhibition of the mTOR pathway, and enhancement of autophagy, confirming the growing interest in the metabolic consequences of energy and carbohydrate restriction. These results are consistent with previous observations that ketogenesis and related changes in lipid and glucose metabolism may lead to improved metabolic parameters in patients with insulin resistance (26, 31). At the same time, the authors highlight the limited knowledge regarding the long-term safety of the ketogenic diet, which remains one of the key research challenges. Interesting results are also presented in the work of Cooper et al. (74), where the ketogenic diet exhibited neuroprotective effects, reducing symptoms of neuropathy associated with metabolic syndrome. This mechanism may be due to the anti-inflammatory properties of ketone bodies and their effects on neuronal metabolism, which is consistent with previous reports of the neuroprotective effects of ketones in other disease entities (2–5). Although this study did not directly address classical metabolic parameters, its results indicate a potentially wider range of benefits of the ketogenic diet in patients with metabolic syndrome, including the nervous system. In the study by Paskalev et al., it has been shown that the ketogenic diet can also be effective in children with obesity and metabolic syndrome, leading to improved insulin resistance and regression of metabolic syndrome symptoms. These results are particularly relevant given the increasing prevalence of obesity and metabolic disorders in the pediatric population. At the same time, however, the use of the ketogenic diet in children requires special caution, due to the risk of nutritional deficiencies and the potential impact on the development of the body (44–46). Further studies are needed to evaluate the safety and long-term efficacy of this intervention in the pediatric population. On the other hand, the results of Chen et al. (29) indicate that the ketogenic diet may improve metabolic parameters such as body weight, glycemia, blood pressure, and lipid profile, as well as reduce inflammation, despite the observed increase in aldosterone. The authors suggest that the ketogenic diet may play a protective role in the context of cardiovascular health, which is consistent with previous reports on the effects of ketogenesis on endothelial function and blood pressure regulation (45, 56). At the same time, however, an increase in aldosterone may be a potential risk factor that requires further research, especially in the context of long-term use of a ketogenic diet. An analysis of the components of the ketogenic diet indicates that it is crucial not only to limit carbohydrates, but also to choose the right fats, proteins and micronutrients. The literature highlights that the dominance of unsaturated fats, especially monounsaturated and polyunsaturated, may support the improvement of lipid profile and the reduction of inflammation (26–28). On the other hand, an excessive supply of saturated fats can lead to an increase in LDL concentration, which is a significant limitation in the context of the prevention of cardiovascular diseases (27). The role of protein in the ketogenic diet is equally important – a moderate supply supports the maintenance of muscle mass, improves satiety and stabilizes glycaemia, but its excess can disrupt ketosis by intensifying gluconeogenesis (34–39). The risk of micronutrient deficiencies, such as magnesium, potassium, sodium, calcium, B vitamins and vitamin D, which play a key role in regulating metabolism, blood pressure and nervous system function, remains an important aspect (42–50). Proper supplementation and proper meal composition are therefore essential for the ketogenic diet to be safe and effective in the long term.

The literature emphasizes that the ketogenic diet, despite its numerous metabolic benefits, also raises controversies regarding cardiovascular safety. Some studies indicate the possibility of an increase in LDL-cholesterol levels and changes in the lipid profile, especially in the case of diets high in saturated fat. This phenomenon is an important argument in the discussion about the long-term safety of ketogenic interventions, especially in patients with pre-existing cardiovascular risk factors. At the same time, the available data are inconclusive, with many studies showing concomitant improvements in other cardiometabolic parameters such as weight reduction, triglyceride reduction, improved insulin resistance, reduced inflammation and blood pressure, which may have a protective effect in the metabolic syndrome population. The studies included in this review did not show a deterioration in key cardiovascular risk indicators; On the contrary, there were improvements in glycaemia, body weight, blood pressure, and lipid parameters, suggesting a potential beneficial effect of the ketogenic diet in this group of patients. However, it should be emphasized that the metabolic response to the ketogenic diet is highly individual and depends, such as on the quality of the fats consumed, the duration of the intervention and the baseline risk profile. For this reason, the use of the ketogenic diet should be carried out in an individualized manner, with regular monitoring of lipid parameters and blood pressure. Current evidence, while promising, remains limited in time, so further long-term studies are needed to evaluate the cardiovascular safety of this intervention in patients with metabolic syndrome.

Overall, the available evidence indicates that the ketogenic diet may provide a valuable tool to support the therapy of metabolic syndrome, leading to improvements in many key metabolic parameters. At the same time, however, its use requires individualization, clinical monitoring, and consideration of potential limitations and side effects. Further, well-designed long-term studies are needed to assess safety, durability and optimal nutritional strategies for different groups of patients with metabolic syndrome.

The interpretation of our findings should also consider the evolution of the definition and pathophysiological understanding of metabolic syndrome. Early conceptualizations, such as Reaven’s seminal description of Syndrome X, emphasized insulin resistance as the central abnormality driving cardiometabolic risk (76). Later frameworks, particularly those proposed by Després and colleagues, highlighted the pivotal role of visceral adiposity and its endocrine activity in amplifying metabolic dysfunction (77, 78). The improvements observed in our review—including reductions in body weight, fasting glucose, triglycerides, and enhanced insulin sensitivity—align with beneficial modulation of all major components described across these definitions. This is consistent with the evidence summarized in our manuscript, where “the ketogenic diet may contribute to weight reduction, improved insulin sensitivity, and beneficial changes in lipid profile, as well as reduced inflammation and improved glycemic control.”

Recent advances in ketogenic interventions further refine their potential applicability in metabolic syndrome. Notably, very-low-calorie ketogenic diets (VLCKD), as outlined in the European Association for the Study of Obesity (EASO) guidelines, demonstrate superior short-term metabolic improvements and structured implementation protocols that may enhance safety and standardization (79). Additionally, the incorporation of medium-chain triglycerides (MCT) has emerged as a promising strategy to improve ketone production, gastrointestinal tolerance, and overall adherence—addressing one of the major limitations highlighted in our review, namely that “concerns remain regarding long-term safety, potential micronutrient deficiencies, and variability in individual responses.” MCT-enriched ketogenic regimens may therefore represent a more feasible and physiologically efficient alternative for selected patients.

Nevertheless, despite these advances, challenges remain regarding the generalizability of ketogenic interventions. Populations with low dietary adherence, limited nutritional support, or comorbidities affecting fat metabolism may not achieve the expected metabolic benefits. As emphasized in our manuscript, “the restrictiveness of the diet makes its long-term use difficult, and safety must be assessed individually.” Future research should therefore prioritize strategies that enhance adherence, evaluate the role of structured VLCKD and MCT-based protocols, and identify patient phenotypes most likely to benefit from ketogenic therapy (76–79).

Future studies on the use of the ketogenic diet in patients with metabolic syndrome should go beyond general efficacy assessments and focus on more precise, clinically relevant areas. First, it is necessary to standardize the protocols of the ketogenic diet, including the proportions of macronutrients, the quality of fats (especially the proportion of saturated vs. unsaturated fats) and the definition of a “well-formulated” ketogenic diet, which will allow the results to be comparable between studies. Second, studies are needed to evaluate cardiovascular safety in different subgroups of patients, especially in those at high baseline risk, taking into account changes in LDL subfractions, inflammatory markers, and endothelial function. Another important direction is to assess the long-term durability of the effects after the end of the ketogenic intervention, including the risk of weight recurrence, metabolic changes, and the impact on eating behavior. It is also worth conducting research on the effects of the ketogenic diet on the gut microbiota, as changes in its composition can modulate metabolic response and inflammation. In addition, future work should seek to identify metabolic phenotypes and biomarkers that can predict which patients benefit most from a ketogenic intervention and who are at higher risk of adverse events. Finally, comparative studies comparing the ketogenic diet with other nutritional interventions used in the metabolic syndrome, such as the Mediterranean diet or the DASH diet, are needed to determine the relative effectiveness and safety of each approach. Such targeted research will allow for a better understanding of both the therapeutic potential and the limitations of the ketogenic diet in this population.

## 6 Limitations and future research

Research on the effects of the ketogenic diet on metabolic syndrome has significant limitations that make it difficult to unequivocally assess its effectiveness. They often include small groups of participants, which limits the ability to generalize the results to the entire population. In addition, many studies take a short time, making it difficult to assess the long-term effects and safety of such a diet. The results also vary due to differences in the composition of the ketogenic diet used in individual studies. Another important problem is the lack of standardization of methods for assessing metabolic parameters, which makes it difficult to compare results. Finally, some studies do not take into account factors such as physical activity or individual genetic predispositions, which can significantly affect the course of metabolic syndrome.

## 7 Conclusion

Studies indicate that restricting carbohydrates in the keto diet improves insulin sensitivity, lowers triglyceride levels, and promotes the reduction of visceral fat, which is especially important in the context of metabolic syndrome. In the short and medium term, this diet can lead to improved metabolic parameters such as glycaemia or lipid profile. However, its effectiveness and safety in the long term have not yet been clearly confirmed, and the results of studies can be mixed. It is worth noting that the use of the ketogenic diet should be done under the care of a dietitian or doctor to avoid deficiencies and monitor possible side effects. In conclusion, the keto diet can be a helpful tool in the fight against metabolic syndrome, but it is not a one-size-fits-all solution and requires an individual approach.
